# Editorial: Innovative approaches in chemotherapy and immunotherapy for gastroenteropancreatic neuroendocrine carcinoma

**DOI:** 10.3389/fonc.2026.1781551

**Published:** 2026-04-27

**Authors:** Evan Vosburgh, Mario Seradilla-Martín, Ho-Man Yeung

**Affiliations:** 1 VA Connecticut Healthcare System, Veterans Health Administration, West Haven, CT, United States; 2 Department of Surgery, Hospital Universitario Virgen de las Nieves, Instituto de Investigación Biosanitaria Ibs. GRANADA, School of Medicine, University of Granada, Granada, Spain; 3 Lewis Katz School of Medicine, Temple University, Philadelphia, PA, United States

**Keywords:** carcinoid, GEPNETs, neuroendocrine, neuroendocrine carcinoma (NEC), rare tumors

Gastroenteropancreatic neuendocrine tumors (GEPNETs) share a common cell of origin, the neuroendocrine cell, diffusely spread throughout the body. They represent a group with numerous subtypes whose primaries arise in numerous locations and tissues, have widely differing presentations, diagnostic approaches and benign to dire prognoses. Though sharing a common cell type, many subtypes of GEPNETs have defied “lumping” in efforts to better understand their basic biology, clinical characterization, or to organize large clinical trials to define diagnostic and therapeutic approaches. Of all GEPNETs, neuroendocrine carcinoma (GEPNEC) is the most aggressive. GEPNEC is a rare and highly aggressive cancer with a poor prognosis and rapid disease progression, comprising less than 1% of all digestive system malignancies. Data indicate that staging, primary tumor site, and histology may be associated with the prognosis of GEPNEC.

The Research Topic *“Innovative Approaches in Chemotherapy and Immunotherapy for Gastroenteropancreatic Neuroendocrine Carcinomas”* highlights efforts to address the challenge of understanding GEPNECs through various approaches. Liu et al. provide a case report and detailed literature review of the fascinating glucagonomas to expand the clinical understanding and avoid the often-delayed diagnosis of these tumors. Wang et al. present a case report and literature review of the rarest of tumors, gallbladder small cell neuroendocrine carcinoma. Using a retrospective approach, Luo et al. provide a review of 31 cases of gallbladder (non-small cell) NEC gathered over 10 years at a single institution. These tumors arise in the gallbladder, a tissue without the common diffuse neuroendocrine cell in the normal state. These studies highlight the process of factors causing metaplasia or reprogramming, a process that might be relevant to other more common GEPNECs, such as the multifocal small bowel GEPNECs. Wu et al. approached the challenge of study of rare tumors through machine learning of a large datasets (SEER and COSMIC) of cases over 19 years of high-grade neuroendocrine colorectal carcinomas. A prediction model derived from 714 patients defined clinical factors for overall survival and mapped the genetic landscape in a way that could guide future analysis and trial design. Mohamed et al. utilized the US National Cancer Database (NCDB) to address the role of surgical cytoreduction in metastatic GEPNETs -on the background of smaller studies and clinical practices supporting surgical approaches. Their study provides strong evidence for a survival benefit for surgical cytoreduction over chemotherapy across many presentations. A close reading of the included manuscripts, though acknowledging the limitations of retrospective design and lack of granularity and incomplete details of large datasets, will provide important advances in understanding these rare tumors, planning further studies, and approaching clinical decision making for often challenging cases.

